# Neurotensin Regulates Proliferation and Stem Cell Function in the Small Intestine in a Nutrient-Dependent Manner

**DOI:** 10.1016/j.jcmgh.2021.09.006

**Published:** 2021-09-22

**Authors:** Stephanie A. Rock, Kai Jiang, Yuanyuan Wu, Yajuan Liu, Jing Li, Heidi L. Weiss, Chi Wang, Jianhang Jia, Tianyan Gao, B. Mark Evers

**Affiliations:** 1 Department of Toxicology and Cancer Biology; 2 Department of Physiology; 3 Markey Cancer Center; 4 Department of Surgery, University of Kentucky, Lexington, Kentucky

**Keywords:** Neurotensin, Intestinal Stem Cells, Diet, *Drosophila*, cDNA, complementary DNA, CRC, colorectal cancer, EdU, 5-ethynyl-2′-deoxyuridine, EGFP, enhanced green fluorescent protein, EE, enteroendocrine, ERK, extracellular-signal-regulated kinase, FACS, fluorescence-activated cell sorting, FAO, fatty acid oxidation, GLP-2, glucagon-like peptide 2, GSEA, gene set enrichment analysis, GSK3β, glycogen synthase kinase 3 beta, ISC, intestinal stem cell, LED, low-energy diet, Lgr5, Leucine-rich repeat-containing G-protein coupled receptor 5, LRP6, low density lipoprotein receptor-related protein 6, MAPK, mitogen-activated protein kinase, mRNA, messenger RNA, NT, neurotensin, NTR1, neurotensin receptor 1, p-AKT, phosphorylated AKT, PBS, phosphate-buffered saline, p-ERK1/2, phosphorylated ERK1/2, p-GSK3β, phosphorylated GSK3β, PPARδ, peroxisome proliferator-activated receptor δ, qPCR, quantitative polymerase chain reaction, TK, tachykinin

## Abstract

**Background & Aims:**

Intestinal stem cells (ISCs) are sensitive to dietary alterations and nutrient availability. Neurotensin (NT), a gut peptide localized predominantly to the small bowel and released by fat ingestion, stimulates the growth of intestinal mucosa under basal conditions and during periods of nutrient deprivation, suggesting a possible role for NT on ISC function.

**Methods:**

Leucine-rich repeat-containing G-protein coupled receptor 5-Enhanced Green Fluorescent Protein (Lgr5-EGFP) NT wild type (*Nt*^*+/+*^) and Lgr5-EGFP NT knockout (*Nt*^*-/-*^*)* mice were fed ad libitum or fasted for 48 hours. Small intestine tissue and crypts were examined by gene expression analyses, fluorescence-activated cell sorting, Western blot, immunohistochemistry, and crypt-derived organoid culture. *Drosophila* expressing NT in midgut enteroendocrine cells were fed a standard diet or low-energy diet and *esg*-green fluorescent protein^+^ ISCs were quantified via immunofluorescence.

**Results:**

Loss of NT impaired crypt cell proliferation and ISC function in a manner dependent on nutrient status. Under nutrient-rich conditions, NT stimulated extracellular signal-regulated kinases 1 and 2 signaling and the expression of genes that promote cell-cycle progression, leading to crypt cell proliferation. Under conditions of nutrient depletion, NT stimulated WNT/β-catenin signaling and promoted an ISC gene signature, leading to enhanced ISC function. NT was required for the induction of WNT/β-catenin signaling and ISC-specific gene expression during nutrient depletion, and loss of NT reduced crypt cell proliferation and impaired ISC function and *Lgr5* expression in the intestine during fasting. Conversely, the expression of NT in midgut enteroendocrine cells of *Drosophila* prevented loss of ISCs during nutrient depletion.

**Conclusions:**

Collectively, our findings establish an evolutionarily conserved role for NT in ISC maintenance during nutritional stress. GSE182828

**Summary**

Neurotensin (NT) is required for fasting-induced activation of WNT/β-catenin and intestinal stem cell (ISC) function. NT enhances ISC function in mice and *Drosophila* during nutrient deprivation, suggesting an evolutionarily conserved role for NT in maintenance of ISCs during nutritional stress.

The intestinal mucosa is in a continuous state of proliferation with complete epithelial cell turnover occurring every 4–5 days.^1^ This rapid self-renewal process is maintained by intestinal stem cells (ISCs) that reside at the base of intestinal crypts.^2^ ISCs give rise to progenitor cells that rapidly proliferate within the crypt and terminally differentiate as they progress up the crypt–villus axis. Progenitor cells differentiate into either absorptive (enterocytes) or secretory (goblet, Paneth, enteroendocrine [EE]) cells that perform the primary functions of the intestinal epithelium, including nutrient absorption, digestion, and protection from microbial infections.^1^ The process of proliferation and differentiation within the small intestine is highly sensitive to nutrient state. Nutrient deprivation causes atrophy of the intestinal mucosa, reduces crypt cell proliferation, and enhances crypt and villus cell apoptosis.^3^ In contrast, ISC function is enhanced by short-term fasting, and ISC numbers are increased by long-term calorie restriction.^4^^,^^5^ Proliferation and stem cell function in the small intestine are controlled by signaling pathways that are tightly coupled to metabolic status, including mitogen-activated protein kinase (MAPK) and WNT/β-catenin signaling.^2^^,^^6^^,^^7^

WNT/β-catenin signaling is an evolutionarily conserved pathway that plays a critical role in intestinal homeostasis.^8^ WNT/β-catenin signaling is activated by translocation of β-catenin to the nucleus, where it activates expression of WNT target genes that regulate cell proliferation, embryonic development, and tissue homeostasis.^9^^,^^10^ Reduction of WNT/β-catenin signaling in the intestine impairs crypt cell proliferation and ISC function, whereas hyperactivation of the WNT/β-catenin pathway contributes to neoplasia.^9^^,^^11^ WNT ligands secreted from subepithelial mesenchymal cells and Paneth cells maintain high levels of WNT/β-catenin signaling at the base of intestinal crypts to support rapid proliferation and ISC self-renewal.^12^^,^^13^ In the mammalian small intestine, active ISCs positioned at the base of crypts are specifically labeled by expression of the WNT target gene *Lgr5*.^14^ Likewise, the *Drosophila* midgut epithelium is replenished by a population of ISCs regulated, in part, by expression of the *Drosophila* WNT homolog, *wingless*.^8^ The WNT/β-catenin pathway is sensitive to nutrient state and metabolic status. Excess dietary fat intake activates WNT/β-catenin signaling in intestinal crypts, leading to enhanced stemness and tumorigenicity of ISCs and progenitor cells.^15^ Free fatty acids increase β-catenin target gene expression in intestinal organoids, and high glucose levels are required for WNT-stimulated nuclear translocation of β-catenin in EE cells and various tumor-derived cell lines.^15^^,^^16^

Nutrient state is also a critical regulator of intestinal hormone release.^17^ The peptide hormone neurotensin (NT) is localized to EE cells predominantly in the distal small intestine.^18^ NT, released primarily by ingestion of dietary fats, facilitates intestinal mucosal proliferation, free fatty acid absorption, lipid metabolism, and glucose homeostasis.^19^^,^^20^ The well-characterized physiologic effects of NT are primarily endocrine-mediated; however, NT is also known to act in a paracrine and autocrine fashion in some cell types, such as certain cancers and intestinal cells.^21^, ^22^, ^23^ NT signaling in the small intestine is mediated predominantly by the G-protein–coupled NT receptor 1 (NTR1), which leads to the activation of growth-stimulating pathways including phosphoinositide 3-kinase (PI3K)/Ak strain transforming(AKT) and MAPK signaling.^24^ Exogenous NT stimulates growth of the normal intestinal mucosa,^25^ contributes to adaptation after intestinal injury,^26^ and has an anti-apoptotic role in intestinal epithelial cells and hepatocytes.^27^ NT has been implicated in the regulation of the WNT/β-catenin pathway in glioblastoma and hepatocellular carcinoma and is a downstream target of the WNT pathway in neuroendocrine tumor cells.^28^, ^29^, ^30^ In colorectal cancer (CRC) cells, NT mediates phosphorylation and inhibition of glycogen synthase kinase 3 beta (GSK3β), a repressor of WNT/β-catenin signaling.^31^ Whether the intestinotrophic effect of NT is mediated through the regulation of ISCs is not known. Here, we show that NT differentially regulates proliferation and stem cell function in the small intestine based on nutrient status. Importantly, our findings show that NT contributes to the maintenance of intestinal homeostasis through regulation of ERK1/2 (in the fed state) and WNT/β-catenin signaling (during nutrient deprivation), and identify an evolutionarily conserved role for NT in the maintenance of ISCs during nutritional stress.

## Results

### NT Deficiency Impairs Intestinal Crypt Cell Proliferation

**Figure 1:**
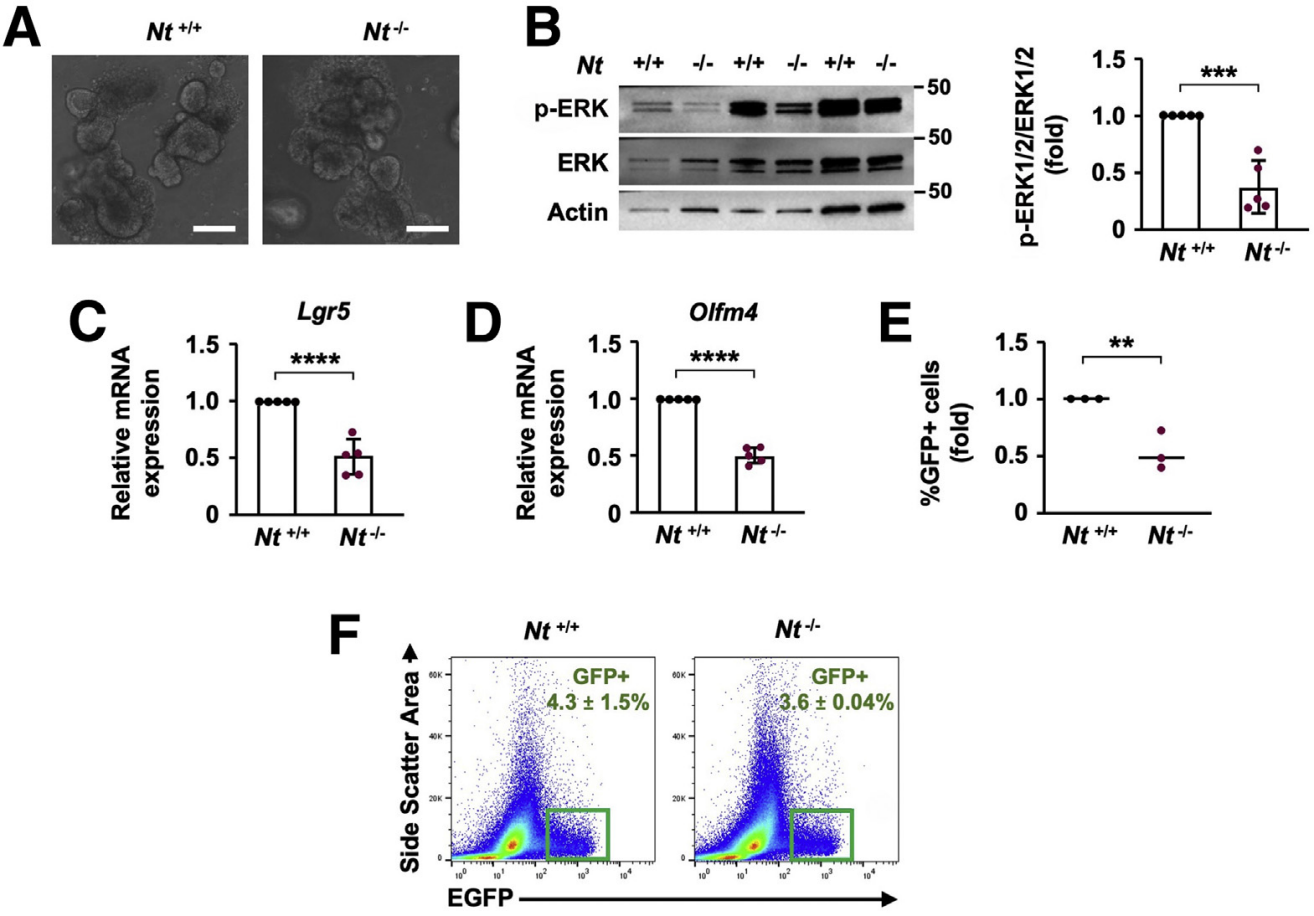
**Loss of NT inhibits ERK1/2 and ISC number in intestinal organoids.** (A) Representative images of organoids from *Nt*^*+/+*^ and *Nt*^*-/-*^ crypts. (*B*) Western blot analysis of p-ERK1/2 and total ERK1/2 in organoids from *Nt*^*+/+*^ and *Nt*^*-/-*^ organoids; organoids from n = 3 mice per group are shown. Densitometry analysis was performed on Western blots of organoids from n = 5 mice per group. (*C*) *Lgr5* and (*D*) *Olfm4* mRNA expression in organoids cultured from *Nt*^*+/+*^ and *Nt*^*-/-*^ crypts; n = 5 mice per group. (*E*) Lgr5-GFP^+^ ISC frequency in organoids cultured from *Nt*^*+/+*^ and *Nt*^*-/-*^ crypts; n = 3 mice per group. (*F*) Lgr5-GFP^+^ ISC frequency in freshly isolated crypts from *Nt*^*+/+*^ and *Nt*^*-/-*^ mice fed ad libitum; n = 3–4 mice per group. Data shown are means ± SD. ∗∗*P* < .01, ∗∗∗*P* < .001, and ∗∗∗∗*P* < .0001. *Scale bars*: 100 μm.

We first investigated the impact of NT on the intestinal epithelium using Leucine-rich repeat-containing G-protein coupled receptor 5-Enhanced Green Fluorescent Protein (Lgr5-EGFP) NT wild type (*Nt*^*+/+*^) and Lgr5-EGFP NT knockout (*Nt*^*-/-*^) mice. NT activates the MAPK family members extracellular signal-regulated kinases 1 and 2 (ERK1/2) in multiple cancer cell lines, including CRC.^32^ The ERK1/2 pathway is a master regulator of the cell cycle, and MAPK/ERK signaling mediates ISC and crypt cell proliferation in the small intestine.^2^^,^^33^ To determine whether NT contributes to ERK1/2 signaling or stemness in the small intestine, we cultured organoids from *Nt*^*+/+*^ and *Nt*^*-/-*^ mice to examine ERK1/2 activation and ISC number. NT deficiency did not alter the appearance of intestinal organoids (Figure 1*A*); however, the absence of NT reduced phosphorylated ERK1/2 (p-ERK1/2) expression by approximately 50% (Figure 1*B*), which corresponded to an approximate 50% decrease in expression of the ISC markers *Lgr5* and *Olfm4* compared with *Nt*^*+/+*^ organoids (Figure 1*C* and *D*). Fluorescence-activated cell sorting (FACS) analysis showed that NT deficiency reduced the percentage of Lgr5-GFP^+^ ISCs in intestinal organoids by approximately 50% relative to intestinal organoids from *Nt*^*+/+*^ mice (Figure 1*E*), indicating that NT plays a role in maintenance of ISC number. However, the number of Lgr5-GFP^+^ ISCs in freshly isolated crypts was unaltered by the absence of NT (Figure 1*F*), suggesting that other factors in the ISC niche likely compensate for the loss of NT in vivo.

**Figure 2:**
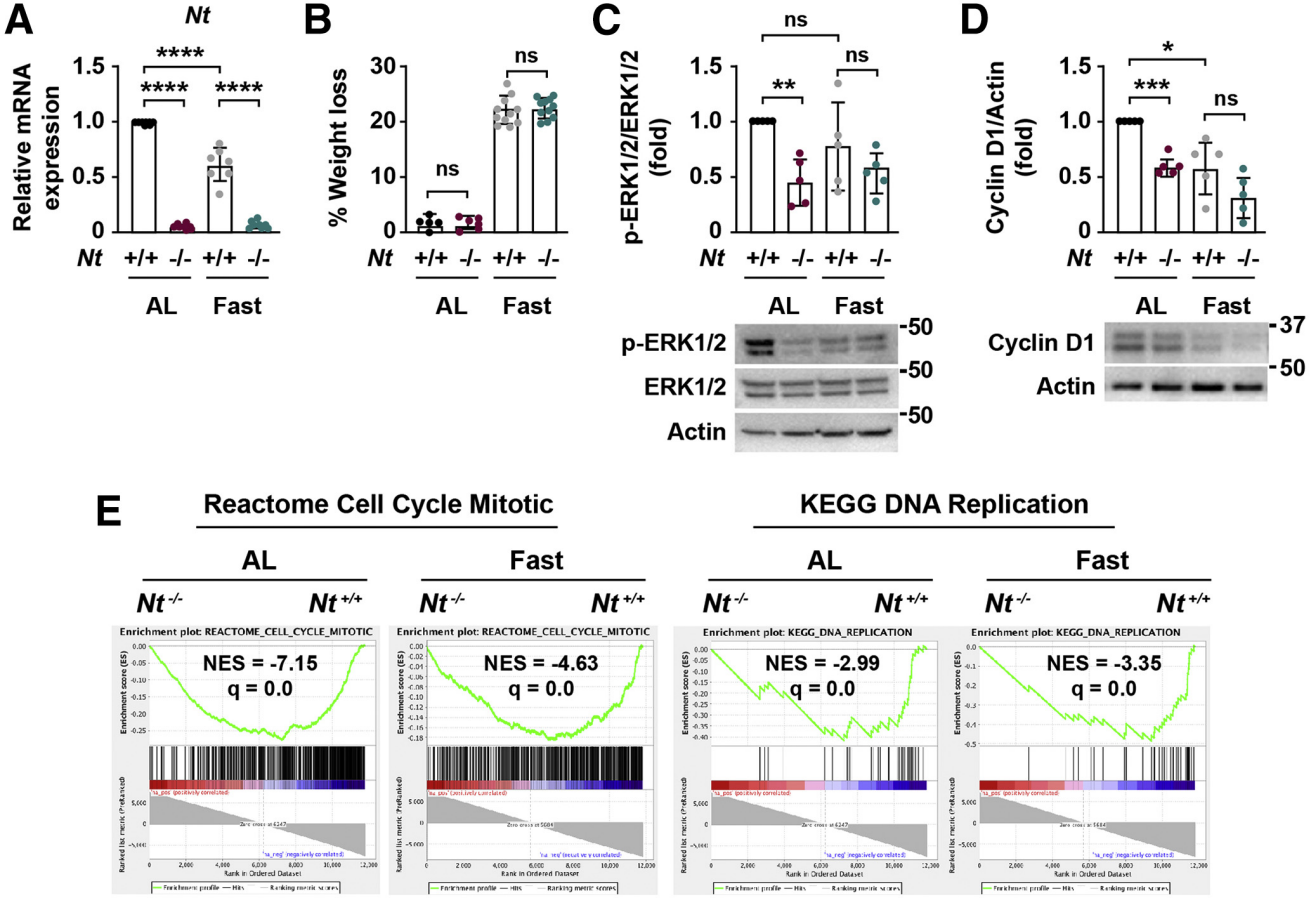
**Loss of NT inhibits ERK1/2 signaling and cell proliferation pathways.** (*A*) *Nt* mRNA expression in crypts isolated from *Nt*^*+/+*^ and *Nt*^*-/-*^ mice fed ad libitum or fasted for 48 hours; n = 7–8 mice per group. (*B*) Body weight of *Nt*^*+/+*^ and *Nt*^*-/-*^ mice fed ad libitum or fasted for 48 hours; n = 5–11 mice per group. (*C*) Representative Western blot of p-ERK1/2 and total ERK1/2 in intestinal crypts isolated from *Nt*^*+/+*^ and *Nt*^*-/-*^ mice fed ad libitum or fasted for 48 hours. *Top*: p-ERK1/2 expression was quantified relative to total ERK1/2 expression in n = 5 mice per group. (*D*) Representative Western blot of cyclin D1 expression in intestinal crypts isolated from *Nt*^*+/+*^ and *Nt*^*-/-*^ mice fed ad libitum or fasted for 48 hours. *Top*: Cyclin D1 expression was quantified relative to actin in n = 5 mice per group. (*E*) Enrichment plots generated by GSEA for Cell Cycle Mitotic and DNA Replication gene sets from the REACTOME and KEGG databases based on RNA sequencing data from crypts isolated from *Nt*^*+/+*^ and *Nt*^*-/-*^ mice fed ad libitum or fasted for 48 hours. Data shown are means ± SD. ∗*P* < .05, ∗∗*P* < .01, ∗∗∗*P* < .001, and ∗∗∗∗*P* < .0001. AL, ad libitum; NES, normalized enrichment score relative to *Nt*^*+/+*^ groups; q, false-discovery rate–adjusted *P* value.

The reduction of ERK1/2 signaling and ISC number in *Nt*^*-/-*^ organoids prompted us to ask whether NT contributes to these functions in vivo under nonhomeostatic conditions. Under conditions of nutritional stress, including acute fasting or long-term calorie restriction, crypt proliferation is impaired and ISC function is altered.^34^ As an intestinotrophic hormone released in response to nutrient ingestion, we hypothesized that NT may contribute to intestinal adaptation depending on nutrient state (ie, fed or fasted). To investigate the role of NT in the small intestine during nutritional stress, *Nt*^*+/+*^ and *Nt*^*-/-*^ mice were fed standard chow ad libitum or fasted for 48 hours. We confirmed loss of NT by evaluating *Nt* messenger RNA (mRNA) from intestinal crypts (Figure 2*A*). Both *Nt*^*+/+*^ and *Nt*^*-/-*^ mice lost approximately 20% of their body weight compared with ad libitum–fed mice; there was no difference in body weight between fasted *Nt*^*+/+*^ and *Nt*^*-/-*^ mice (Figure 2*B*).

NT has been shown to promote ERK1/2 signaling in various CRC cell lines in vitro, yet whether NT regulates ERK1/2 signaling in the small intestine in vivo is not known. In organoids from *Nt*^*-/-*^ mice, p-ERK1/2 expression was down-regulated relative to *Nt*^*+/+*^ organoids (Figure 1*B*), indicating that NT promotes ERK1/2 signaling in intestinal cells ex vivo. To determine whether NT regulates the ERK1/2 pathway in vivo, we measured p-ERK1/2 in crypts isolated from ad libitum and fasted *Nt*^*+/+*^ and *Nt*^*-/-*^ mice. Consistent with our findings in *Nt*^*-/-*^ organoids, the absence of NT reduced p-ERK1/2 expression in the crypts by approximately 50% relative to ad libitum–fed controls, although p-ERK1/2 expression was not altered in the crypts of fasted *Nt*^*-/-*^ mice (Figure 2*C*). In agreement with these data, expression of the ERK1/2 target, cyclin D1, was reduced by approximately 50% in crypts from *Nt*^*-/-*^ mice relative to ad libitum–fed controls (Figure 2*D*). Cyclin D1 expression was reduced to a similar extent by fasting alone but was not reduced further by the absence of NT. To quantify gene expression changes associated with NT during nutrient stress, we performed a bulk RNA sequencing analysis using crypts isolated from ad libitum–fed and fasted *Nt*^*+/+*^ and *Nt*^*-/-*^ mice. Because ERK1/2 and cyclin D1 are critical regulators of cell-cycle progression and proliferation, we performed a gene set enrichment analysis (GSEA) to better delineate the role of NT on pathways regulating the cell cycle and cell proliferation. GSEA showed that absence of NT down-regulated cell-cycle progression, mitosis, and DNA synthesis pathways compared with ad libitum–fed and fasted *Nt*^*+/+*^ mice (Figure 2*E*), providing further evidence that NT positively regulates crypt cell proliferation.

**Figure 3:**
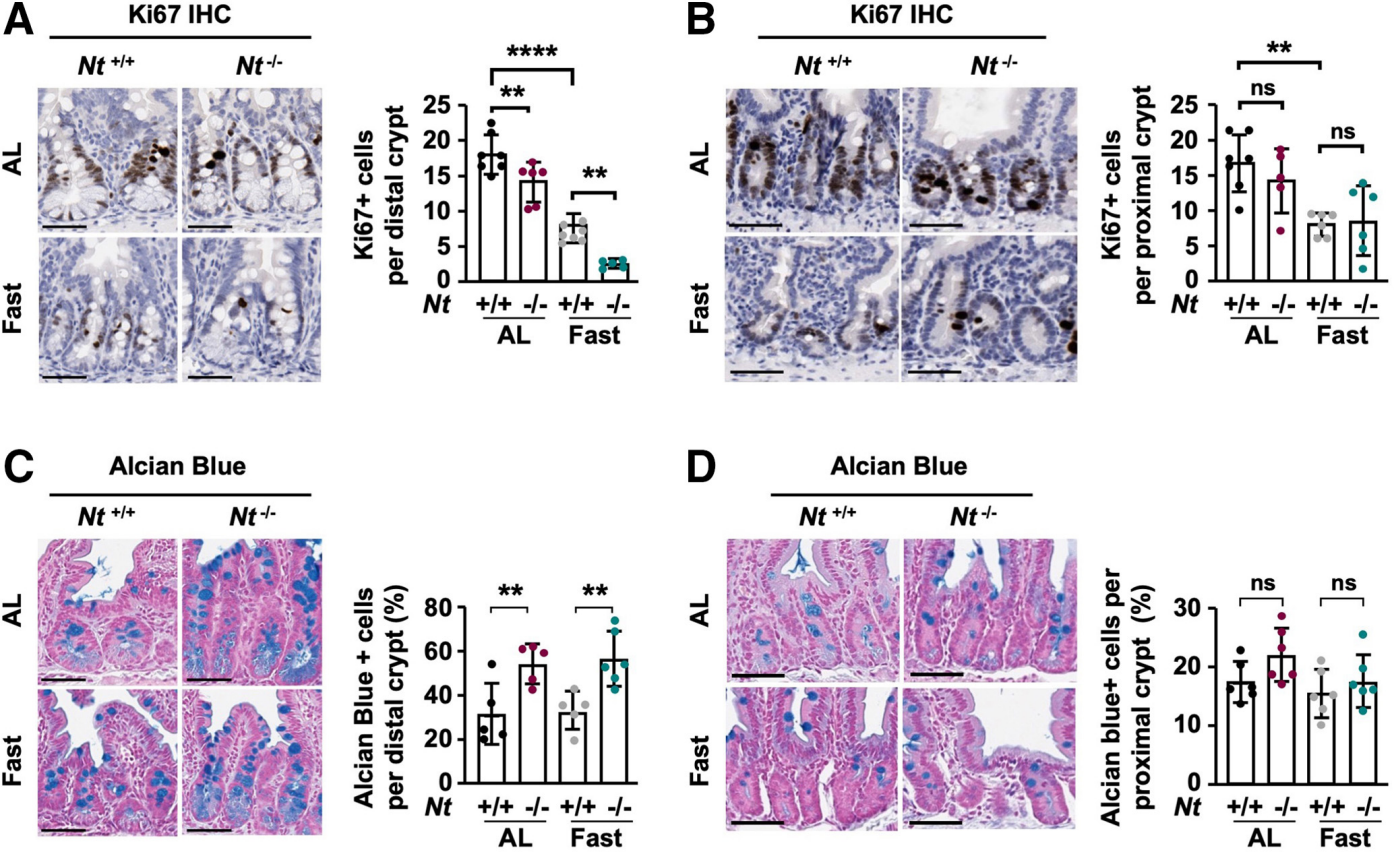
**Loss of NT impairs crypt cell proliferation and promotes differentiation.** (*A*) Representative Ki67 immunohistochemistry in distal intestinal crypts from *Nt*^*+/+*^ and *Nt*^*-/-*^ mice fed ad libitum or fasted for 48 hours. *Right*: Quantification of the average number of Ki67^+^ cells per crypt in 50 crypts per section in n = 6–7 mice per group. (*B*) Representative Ki67 immunohistochemistry in proximal intestinal crypts from *Nt*^*+/+*^ and *Nt*^*-/-*^ mice fed ad libitum or fasted for 48 hours. *Right*: Quantification of the average number of Ki67^+^ cells per in over 50 crypts per section in n = 5–7 mice per group. (*C*) Representative Alcian blue staining in distal intestinal crypts from *Nt*^*+/+*^ and *Nt*^*-/-*^ mice fed ad libitum or fasted for 48 hours. *Right*: Quantification of the percentage of Alcian blue^+^ cells per crypt in 50 crypts per section in n = 5–6 mice per group. (*D*) Representative Alcian blue staining in proximal intestinal crypts from *Nt*^*+/+*^ and *Nt*^*-/-*^ mice fed ad libitum or fasted for 48 hours. *Right*: Quantification of the percentage of Alcian blue^+^ cells per crypt in 50 crypts per section in n = 6 mice per group. Data shown are means ± SD. ∗∗*P* < .01 and ∗∗∗∗*P* < .0001. *Scale bars*: 50 μm. AL, ad libitum.

To validate these results in vivo, we quantified expression of the proliferation marker Ki67^35^ in the proximal and distal small intestine. Consistent with previous studies,^4^^,^^36^ fasting reduced the number of Ki67^+^ cells in the crypts by approximately 50% compared with ad libitum–fed mice in both the proximal and distal small intestine (Figure 3*A* and *B*). Moreover, the absence of NT significantly reduced the number of Ki67^+^ cells in the distal crypts under both ad libitum–fed and fasted conditions relative to *Nt*^*+/+*^ mice (Figure 3*A*). However, NT deficiency did not impact the frequency of Ki67^+^ cells in the proximal small intestine (Figure 3*B*), suggesting that the effect of endogenous NT is greatest in the distal small intestine, where NT expression is highest.^37^ The reduction in proliferating distal crypt cells in *Nt*^*-/-*^ mice corresponded to increased epithelial cell differentiation measured via Alcian blue staining of goblet cells. *Nt*^*-/-*^ approximately doubled the percentage of goblet cells in the distal crypts of ad libitum–fed and fasted mice compared with *Nt*^*+/+*^ mice (Figure 3*C*), whereas no significant changes were observed in goblet cell frequency in the proximal small intestine (Figure 3*D*). Together, these data show that NT contributes to cell proliferation and differentiation in the distal small intestine independent of nutrient status. These effects likely are mediated by NT regulation of ERK1/2 signaling.

### Fasting Activates WNT/β-Catenin Signaling in an NT-Dependent Manner

**Figure 4:**
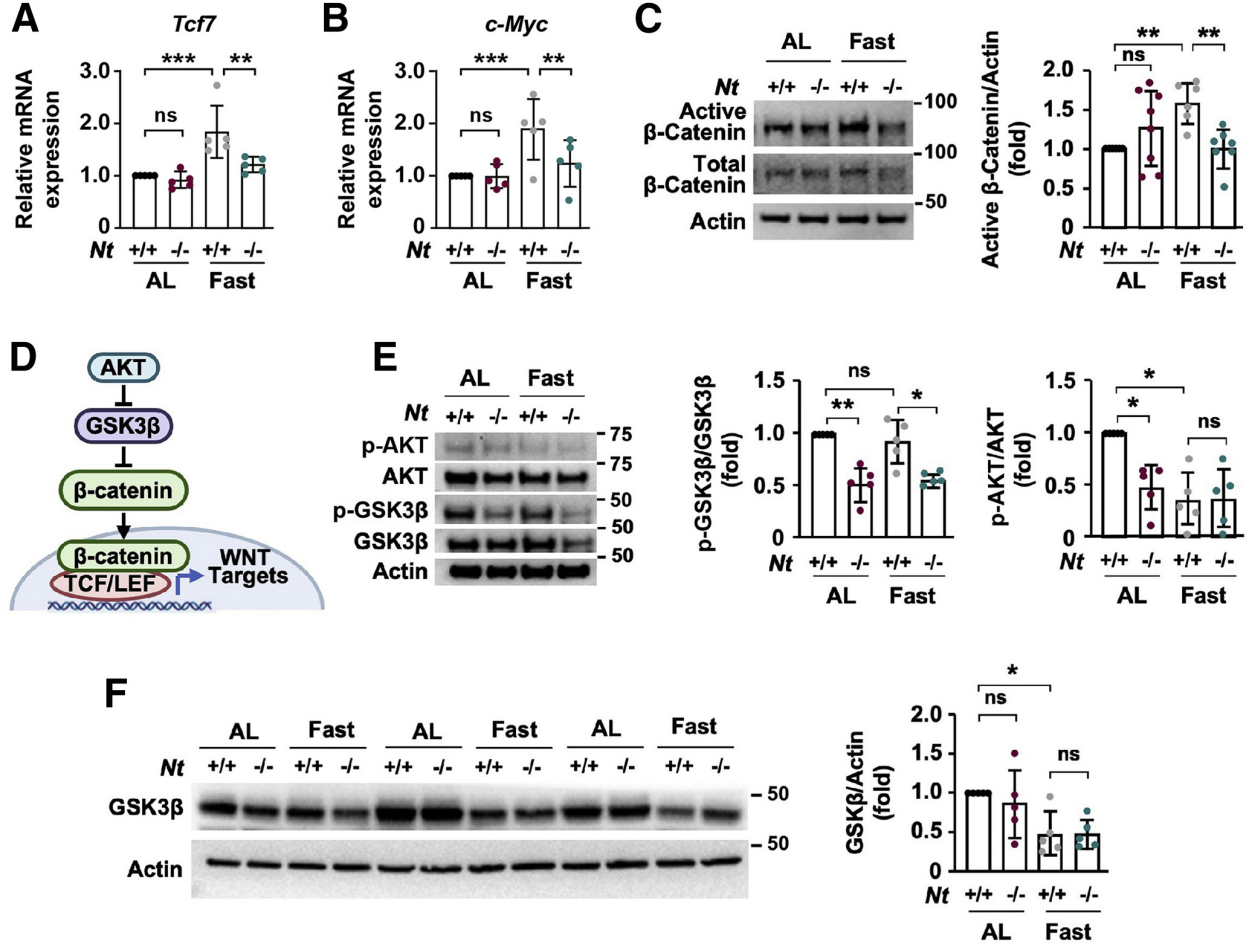
**NT is required for induction of WNT/β-catenin signaling in intestinal crypts during fasting.** Real-time -qPCR analysis of (*A*) *Tcf7* and (*B*) *c-Myc* mRNA expression in RNA from intestinal crypts isolated from *Nt*^*+/+*^ and *Nt*^*-/-*^ mice fed ad libitum or fasted for 48 hours; n = 5 mice per group. (*C*) Representative Western blot of active and total β-catenin expression in intestinal crypts isolated from *Nt*^*+/+*^ and *Nt*^*-/-*^ mice fed ad libitum or fasted for 48 hours. *Right*: Active β-catenin expression was quantified relative to actin expression in n = 6–8 mice per group. (*D*) Schematic of AKT/GSK3β-mediated activation of the WNT/β-catenin pathway. AKT phosphorylates and inhibits GSK3β, leading to β-catenin stabilization and nuclear translocation. (*E*) Representative Western blot of p-AKT and total AKT and p-GSK3β and total GSK3β expression in intestinal crypts isolated from *Nt*^*+/+*^ and *Nt*^*-/-*^ mice fed ad libitum or fasted for 48 hours. *Right*: p-GSK3β and p-AKT expression was quantified relative to total protein expression in n = 5 mice per group. (*F*) Representative Western blot analysis of total GSK3β in crypts from *Nt*^*+/+*^ and *Nt*^*-/-*^ mice fed ad libitum or fasted for 48 hours; n = 3 mice per group. *Right*: Densitometry analysis was performed to quantify total GSK3β expression relative to actin in n = 5 mice per group. Data shown are means ± SD. ∗*P* < .05, ∗∗*P* < .01, and ∗∗∗*P* < .001. AL, ad libitum; TCF/LEF, T cell factor/lymphoid enhancer factor.

Crosstalk between NT/NTR1 and the WNT/β-catenin pathway has been shown in multiple cancer cell lines,^28^, ^29^, ^30^ yet whether NT regulates WNT/β-catenin signaling in the small intestine is unknown. To determine whether NT-mediated effects on intestinal proliferation are associated with alterations in the WNT/β-catenin pathway, we examined expression of the T cell factor/lymphoid enhancer factor (TCF/LEF) transcription factor *Tcf7*, a main effector of WNT/β-catenin signaling,^38^ in crypts from ad libitum–fed and fasted *Nt*^*+/+*^ and *Nt*^*-/-*^ mice. Strikingly, despite reducing crypt cell proliferation, fasting increased *Tcf7* mRNA expression by approximately 80% in *Nt*^*+/+*^ crypts relative to ad libitum–fed controls (Figure 4*A*). Although the absence of NT had no impact on *Tcf7* mRNA in the crypts of ad libitum–fed mice, loss of NT completely abrogated the induction of *Tcf7* mRNA during fasting. We observed similar expression of the WNT target gene *c-Myc*, which was increased by approximately 80% in fasted *Nt*^*+/+*^ crypts but was not increased in the crypts of fasted *Nt*^*-/-*^ mice (Figure 4*B*). In agreement with WNT/β-catenin activation, fasting increased expression of active β-catenin in the crypts from *Nt*^*+/+*^ mice by greater than 50% compared with crypts from ad libitum–fed *Nt*^*+/+*^ mice, but did not increase active β-catenin in the crypts from *Nt*^*-/-*^ mice (Figure 4*C*), suggesting that NT positively regulates WNT/β-catenin signaling during periods of fasting.

To elucidate the mechanism by which NT activates WNT/β-catenin during fasting, we next examined pathways downstream of NT signaling that may converge on the WNT/β-catenin pathway. In CRCs, NT signaling promotes phosphorylation of GSK3β, a negative regulator of WNT/β-catenin signaling.^31^ GSK3β phosphorylates β-catenin on Ser33, Ser37, and Thr41, leading to ubiquitination and proteasomal degradation of β-catenin.^39^ Phosphorylation of GSK3β on Ser9 by AKT inhibits GSK3β activity, thereby stabilizing β-catenin and enabling its nuclear translocation and activation of WNT signaling (Figure 4*D*).^40^, ^41^, ^42^ To determine whether NT promotes WNT/β-catenin signaling through regulation of GSK3β, we examined phosphorylation of GSK3β on Ser9 in crypts isolated from ad libitum–fed and fasted *Nt*^*+/+*^ and *Nt*^*-/-*^ mice. The absence of NT reduced p-GSK3β expression relative to crypts harvested from *Nt*^*+/+*^ mice under both ad libitum–fed and fasted conditions (Figure 4*E*). Although fasting alone did not alter the ratio of phosphorylated and total GSK3β, total GSK3β expression was reduced significantly by fasting (Figure 4*F*), suggesting GSK3β depletion as a potential mechanism for WNT/β-catenin activation during fasting. Consistent with p-GSK3β expression, *Nt*^*-/-*^ reduced p-AKT expression by approximately 50% in the crypts from ad libitum–fed mice (Figure 4*E*). Fasting alone reduced p-AKT to a similar extent; however, no additional decrease of p-AKT was observed in the crypts of fasted *Nt*^*-/-*^ mice. These data show that NT contributes to the induction of WNT/β-catenin signaling in the small intestine during fasting. Moreover, our findings suggest that this induction may occur through NT regulation of the AKT/GSK3β pathway*.*

### Exogenous NT Activates WNT/β-Catenin Signaling in the Small Intestine

**Figure 5:**
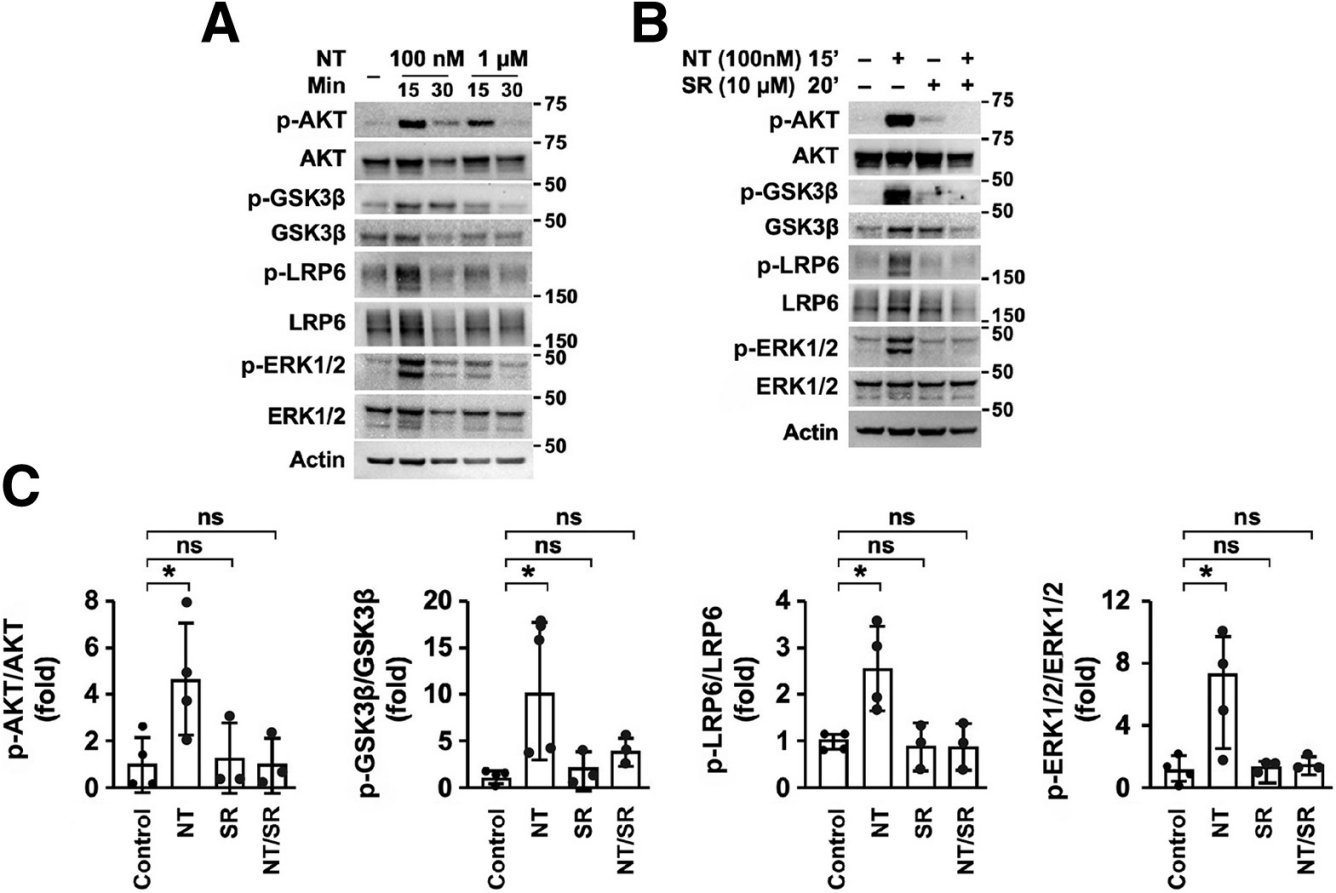
**Exogenous NT activates WNT/β-catenin signaling in the small intestine.** (*A*) Representative Western blot analysis of proteins in the WNT/β-catenin and AKT/GSK3β signaling pathways in full-thickness small intestine treated ex vivo with 100 nmol/L or 1 μmol/L NT for 15 or 30 minutes. (*B*) Representative Western blot analysis of phosphorylated and total forms of AKT, GSK3β, LRP6, and ERK1/2 in full-thickness small intestine treated with NT (100 nmol/L) for 15 minutes, SR48692 (10 μmol/L) for 20 minutes, or pretreated with SR48692 (10 μmol/L) for 20 minutes and treated with NT (100 nmol/L) for 15 minutes. (*C*) Phosphorylated AKT, GSK3β, LRP6, and ERK1/2 expression was quantified relative to total protein expression in n = 3–4 mice per group. Data shown are means ± SD. ∗*P* < 0.05. SR, SR48692; Min, minutes.

We next determined whether exogenous NT activates WNT/β-catenin and AKT/GSK3β signaling in the small intestine. Full-thickness small intestine was isolated from *Nt*^*+/+*^ mice and treated with NT (100 nmol/L or 1 μmol/L) in phosphate-buffered saline (PBS) for 15 or 30 minutes at 37°C. Western blot analysis showed increased AKT phosphorylation after a 15-minute incubation with either 100 nmol/L or 1 μmol/L NT (Figure 5*A*). After 30 minutes of exposure, p-AKT expression remained increased relative to controls, but was decreased relative to the 15-minute time point, indicating that short-term treatment with NT potently activates AKT signaling in the small intestine. Consistent with AKT activation, we found that 15 minutes of exposure to 100 nmol/L NT increased p-GSK3β and phosphorylated LRP6, a marker of WNT/β-catenin activation, by approximately 10- and 2.5-fold, respectively (Figure 5*A* and *C*). In agreement with our initial findings that NT depletion reduces ERK1/2 signaling in crypts, exogenous NT increased phosphorylation of ERK1/2 by approximately 6-fold (Figure 5*A* and *C*).

To further confirm that activation of these pathways was mediated by NT/NTR1 signaling, we treated full-thickness small intestine with 100 nmol/L NT for 15 minutes with or without 20 minutes of pretreatment with the NTR1 inhibitor SR48692.^43^ NT consistently increased phosphorylation of AKT, GSK3β, LRP6, and ERK1/2, whereas pretreatment with SR48692 prevented NT-mediated phosphorylation of these proteins (Figure 5*B* and *C*). Collectively, these data show that NT/NTR1 signaling activates WNT/β-catenin, AKT/GSK3β, and ERK1/2 signaling in the small intestine.

### NT Promotes ISC Gene Expression During Fasting

**Figure 6:**
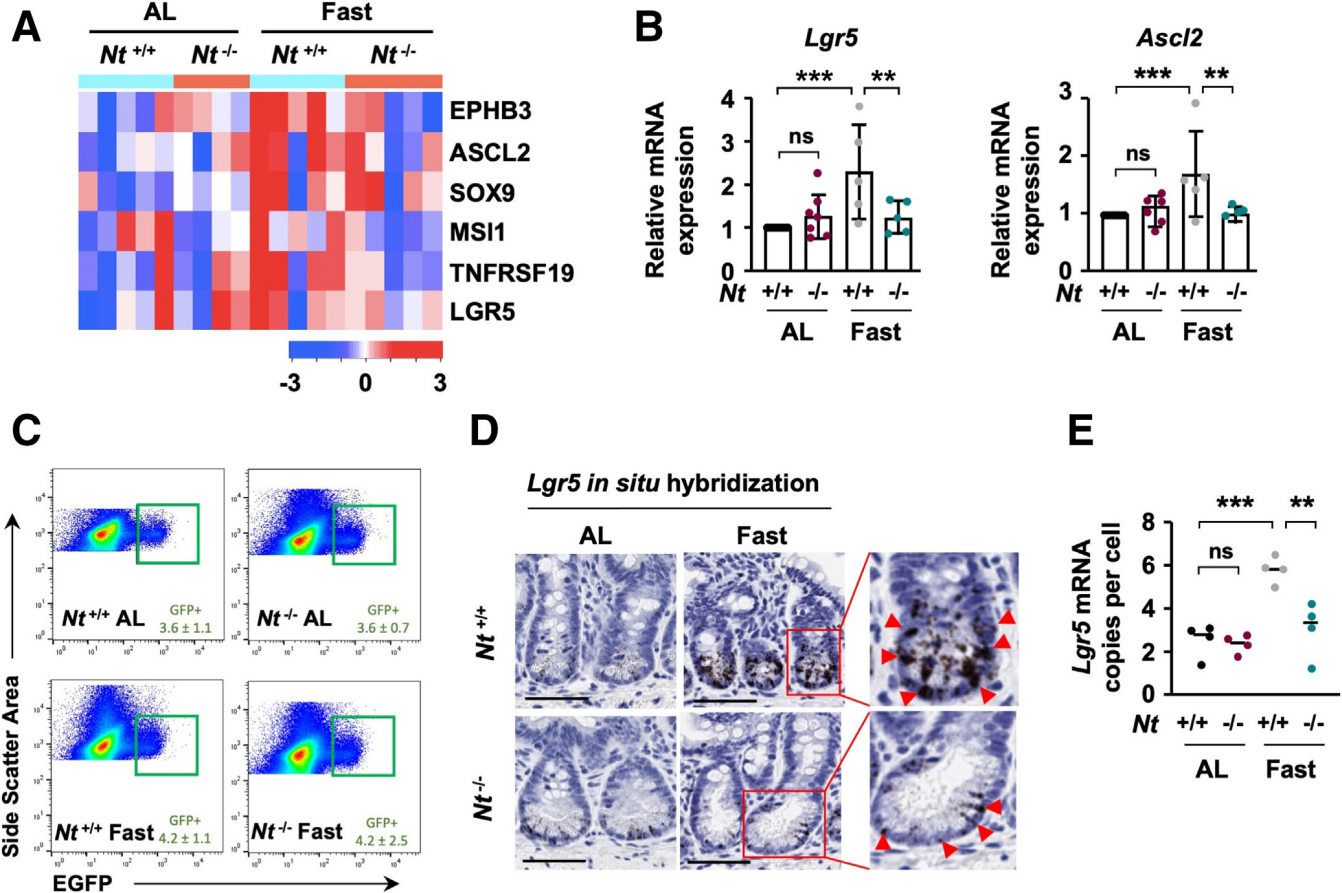
**Loss of NT impairs ISC-specific gene expression during fasting.** (*A*) Heatmap of RNA sequencing analysis showing expression of ISC-specific WNT target genes in crypts isolated from *Nt*^*+/+*^ and *Nt*^*-/-*^ mice fed ad libitum or fasted for 48 hours; n = 5 mice per group for *Nt*^*+/+*^ ad libitum, *Nt*^*+/+*^ Fast, and *Nt*^*-/-*^ Fast groups, n = 4 for *Nt*^*-/-*^ ad libitum group. (*B*) real-time -qPCR analysis of *Lgr5* and *Ascl2* mRNA expression in RNA from intestinal crypts isolated from *Nt*^*+/+*^ and *Nt*^*-/-*^ mice fed ad libitum or fasted for 48 hours; n = 5–8 mice per group. (*C*) Lgr5-GFP^+^ ISC frequency in freshly isolated crypts from *Nt*^*+/+*^ and *Nt*^*-/-*^ mice fed ad libitum or fasted for 48 hours; n = 4 mice per group. (*D*) Representative images of *Lgr5* in situ hybridization in distal crypts from *Nt*^*+/+*^ and *Nt*^*-/-*^ mice fed ad libitum or fasted for 48 hours. *Scale bars*: 50 μm. *Inset*: *Arrowheads* indicate *Lgr5*^+^ cells. (*E*) Quantification of *Lgr5* mRNA copies per cell determined by in situ hybridization; n = 4 mice per group. Data shown are means ± SD. ∗∗*P* < .01, ∗∗∗*P* < .001. AL, ad libitum.

WNT/β-catenin signaling is critical for the proliferation and maintenance of ISCs, which are characterized by their high expression of WNT target genes, including *Lgr5*, *Ascl2*, *Sox9*, *Msi-1*, and *EphB3*.^38^ Consistent with activation of WNT/β-catenin signaling in the crypts of fasted *Nt*^*+/+*^ mice, RNA sequencing analysis suggested up-regulation of ISC-specific genes in the crypts of fasted *Nt*^*+/+*^ mice relative to ad libitum–fed *Nt*^*+/+*^ mice (fold change, >1), although not all of these genes reached statistical significance (false-discovery rate–adjusted *P* value < .05). In contrast, fasting did not up-regulate expression of ISC-specific genes in the crypts of *Nt*^*-/-*^ mice (*P* > .05) (Figure 6*A*). We performed quantitative real-time polymerase chain reaction (real-time qPCR) to validate expression of *Lgr5* and *Ascl2*, which are highly restricted to active ISCs,^2^ and confirmed that fasting significantly up-regulated expression of these ISC markers in *Nt*^*+/+*^ mice but not in *Nt*^*-/-*^ mice (Figure 6*B*), indicating that NT is required for the induction of active ISC markers during fasting.^44^ We next analyzed the frequency of Lgr5^+^ cells in the crypts using FACS. In contrast to the pattern of *Lgr5* mRNA during fasting, neither fasting nor absence of NT altered the total number of Lgr5-GFP^+^ ISCs (Figure 6*C*). To more precisely delineate the localization of *Lgr5* mRNA expression in vivo, we performed single-molecule in situ hybridization in the distal small intestine, where we observed that the effects of NT are most pronounced. In agreement with RNA sequencing and real-time qPCR analyses, fasting increased *Lgr5* mRNA copies per crypt cell by 3-fold relative to ad libitum–fed controls, but did not significantly increase *Lgr5* expression in the crypts of *Nt*^*-/-*^ mice (Figure 6*D* and *E*). Thus, rather than increasing the number of Lgr5^*+*^ ISCs, fasting increased the expression of *Lgr5* in the established population of ISCs and progenitor cells in an NT-dependent manner.

### NT Increases ISC Function During Nutrient Deprivation

**Figure 7:**
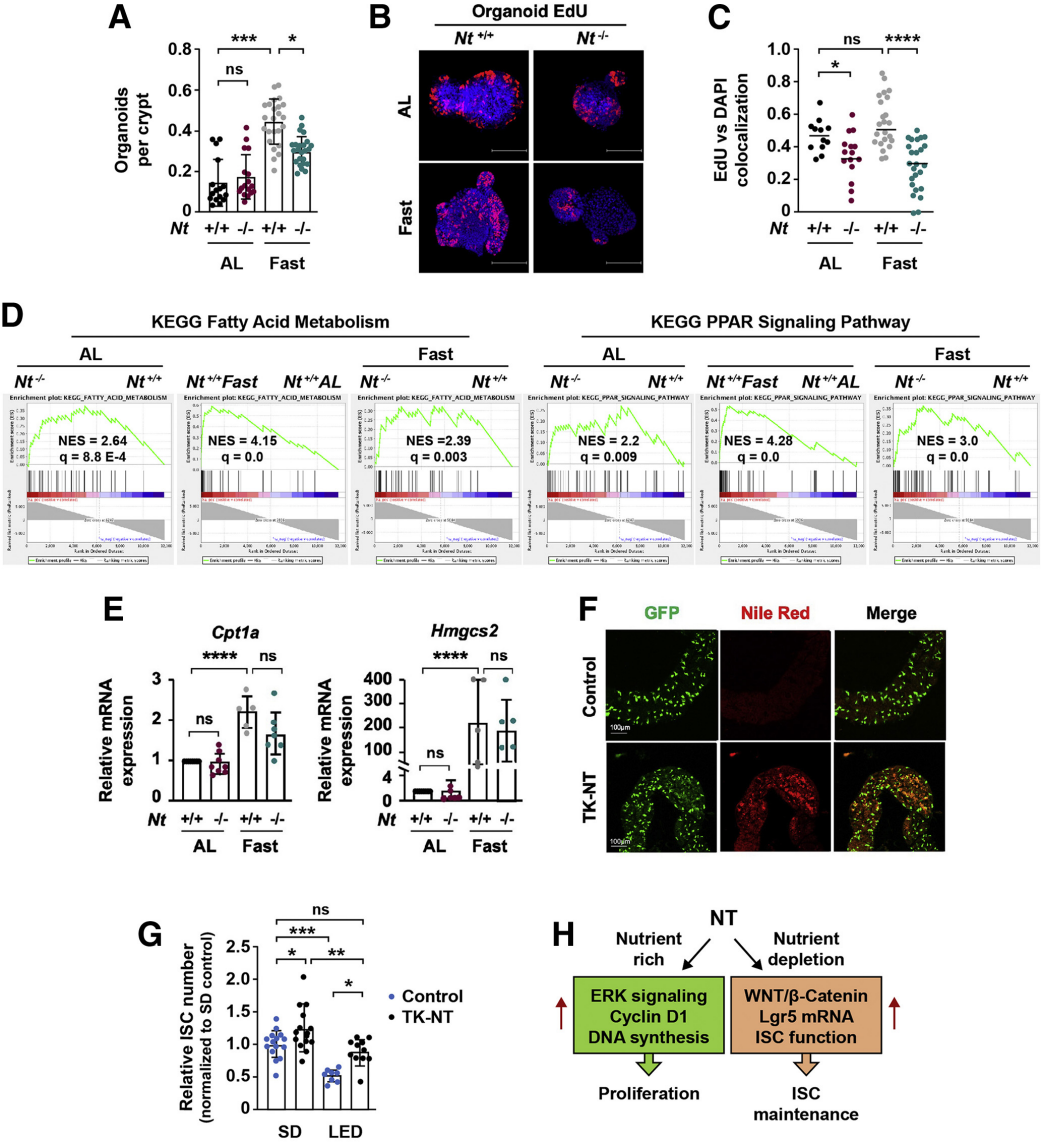
**NT contributes to ISC maintenance during nutrient-stress.** (*A*) Organoid-forming efficiency of crypts from *Nt*^*+/+*^ and *Nt*^*-/-*^ mice fed ad libitum or fasted for 48 hours; n = 5–6 mice per group. (*B*) Representative EdU staining and quantification of EdU^+^ cells (*C*) in primary organoids cultured from *Nt*^*+/+*^ and *Nt*^*-/-*^ mice fed ad libitum or fasted for 48 hours. *Scale bars*: 100 μm. EdU^+^ cells were quantified using the Pearson coefficient of colocalization between EdU and 4',6-diamidino-2-phenylindole in 20–30 organoids from 3 to 4 mice per group. (*D*) Enrichment plots generated by GSEA for Fatty Acid Metabolism and PPAR Signaling gene sets from the KEGG database based on RNA sequencing data from crypts isolated from *Nt*^*+/+*^ and *Nt*^*-/-*^ mice fed ad libitum or fasted for 48 hours. (*E*) real-time qPCR analysis of *Cpt1a* and *Hmgcs2* mRNA expression in RNA from intestinal crypts isolated from *Nt*^*+/+*^ and *Nt*^*-/-*^ mice fed ad libitum or fasted for 48 hours; n = 5–7 mice per group. (*F*) Representative *esg*-GAL4-GFP and Nile Red staining in the midgut of control and NT-expressing (TK-NT) *Drosophila*. (*G*) Quantification of *esg*-GAL4-GFP^+^ ISCs in the midgut of control and NT-expressing (TK-NT) *Drosophila* fed a standard diet or a LED; data represent the number of ISCs in 8–15 flies per group. (*H*) Summary of the nutrient-state–dependent function of NT on crypt cell proliferation and ISC maintenance. Data shown are means ± standard deviation. ∗*P* < .05, ∗∗*P* < .01, ∗∗∗*P* < .001, and ∗∗∗∗*P* < .0001. AL, ad libitum; DAPI, 4',6-diamidino-2-phenylindole; q, false-discovery rate–adjusted *P* value; NES, normalized enrichment score relative to *Nt*^*+/+*^ groups; SD, standard diet.

Fasting has been shown previously to augment ISC function without altering ISC frequency.^4^^,^^36^ To determine whether the NT-dependent increase in *Lgr5* mRNA expression during fasting is associated with changes in ISC function, crypts were isolated from ad libitum–fed and fasted *Nt*^*+/+*^ and *Nt*^*-/-*^ mice for ex vivo colony formation assays. Consistent with others,^4^ we found an approximate 3-fold increase in organoid-forming efficiency in fasted *Nt*^*+/+*^ crypts relative to the crypts from ad libitum–fed *Nt*^*+/+*^ mice (Figure 7*A*). However, organoid formation was reduced significantly in the crypts from fasted *Nt*^*-/-*^ mice compared with *Nt*^*+/+*^ mice. Furthermore, analysis of 5-ethynyl-2′-deoxyuridine (EdU) incorporation, an indicator of cell proliferation,^45^ in crypt-derived organoids showed a significant reduction in EdU uptake in fasted *Nt*^*-/-*^ mice relative to fasted *Nt*^*+/+*^ mice (Figure 7*B* and *C*). We next examined whether NT augments fatty acid oxidation (FAO), which promotes ISC function during fasting via activation of peroxisome proliferator-activated receptor delta (PPARδ) signaling and enhanced function of Cpt1a, the rate-limiting enzyme in FAO.^4^ Interestingly, FAO and PPAR gene sets were enriched in *Nt*^*-/-*^ mice relative to *Nt*^*+/+*^ mice under both ad libitum–fed and fasted conditions (Figure 7*D*), suggesting a role for NT in negative regulation of these pathways. However, no significant changes in the PPARδ target genes *Cpt1a* or *Hmgcs2* were observed between fasted *Nt*^*+/+*^ and fasted *Nt*^*-/-*^ mice (Figure 7*E*), suggesting that the effects of NT on ISC function are not mediated by alterations in FAO. Instead, our findings indicate that NT increases ISC function during fasting via up-regulation of WNT target genes (ie, *Lgr5*) in ISCs and progenitor cells.

Regulation of the intestinal epithelium is highly conserved, such that similar signaling pathways and cellular processes regulate intestinal homeostasis in the mammalian and *Drosophila* intestine.^46^ The *Drosophila* midgut is composed of ISCs that differentiate into the same major lineages as those in the mammalian small intestine, and ISC proliferation is regulated by expression of the *Drosophila* WNT homolog, *wingless*.^46^ Therefore, the *Drosophila* ISC reporter line *esg*-Gal4-GFP, in which GFP is fused to the *Drosophila* ISC marker *escargot*, is a powerful model for studying ISC function.^47^ We previously identified CG9918 (pyrokinin 1 receptor) as the endogenous NTR-like receptor in *Drosophila*.^20^ NT expression in the *Drosophila* midgut mimics the effects of NT on intestinal lipid absorption and adenosine monophosphate-activated protein kinase activation in the mammalian small intestine, suggesting that the effects of NT on the intestinal epithelium are highly conserved.^20^ To further delineate the role of NT on ISC function, we expressed human full-length NT complementary DNA (cDNA) in midgut EE cells using the EE cell-specific tachykinin (TK) promoter and examined ISCs labeled by *esg*-Gal4-GFP (*esg*-GFP) (Figure 7*F*).^48^^,^^49^ *esg*-Gal4-GFP TK-NT and *esg*-Gal4-GFP control flies were maintained for 5 days on a standard diet or a nutrient-reduced low-energy diet (LED).^50^^,^^51^ Consistent with our previous findings,^20^ NT overexpression increased the accumulation of lipid droplets in *Drosophila* midgut measured by Nile Red staining (Figure 7*F*, center panels). In flies fed a standard diet, NT expression caused a slight but significant increase in *esg*-GFP^+^ ISCs relative to control flies (Figure 7*G*). In flies fed a LED, the frequency of *esg*-GFP^+^ ISCs was reduced by more than 50% compared with flies fed a standard diet. However, expression of NT in flies fed a LED restored *esg*-GFP^+^ ISC numbers to that of control flies fed a standard diet (Figure 7*G*). These data indicate that NT increases ISC number in *Drosophila* midgut and attenuates ISC depletion during nutrient deprivation. Collectively, our findings show that NT contributes to the maintenance of ISCs in both mice and *Drosophila* during periods of nutrient deprivation, thus indicating an evolutionarily conserved function of NT on ISCs.

## Discussion

The essential processes of digestion and nutrient absorption that take place in the small intestine are dependent on the constant proliferation and differentiation of intestinal epithelial cells.^1^ This continuous self-renewal process is mediated by ISCs, which integrate dietary signals to maintain intestinal equilibrium in an environment of constant nutrient flux.^52^ Our study identified a novel and evolutionarily conserved role for NT, acting through NTR1, in the nutrient-dependent regulation of ISC function. We found that NT contributes to ISC maintenance in mice during nutritional stress and activated AKT/GSK3β and WNT/β-catenin signaling in the small intestine. In a complementary study, we showed that NT expression increased ISC number in the midgut of *Drosophila* fed a standard diet and prevented ISC loss during nutrient depletion. Consistent with our findings in the normal intestinal mucosa, NT has been shown to promote stem cell function and WNT/β-catenin signaling in hepatocellular cancers^30^^,^^53^ and enhance stem cell–like traits in glioblastoma cells.^54^ Given the known proliferative effects of NT on CRC cells, it will be interesting to determine whether NT contributes to stem cell function in CRC stem cells, particularly in the context of a high-fat diet. Previous work in *Drosophila* underscores the importance of intestinal hormones in the regulation of ISC homeostasis.^48^ Deletion of EE cells in *Drosophila* midgut prevents nutrient-stimulated expression of the *Drosophila* insulin-like peptide 3 and impairs ISC proliferation, indicating that EE cells are important nutrient-sensing modulators of ISC homeostasis.^48^ Importantly, our findings support a model in which a gut hormone–ISC axis mediates ISC adaptation to nutritional stress.

Consistent with the protective function of NT on ISCs, glucagon-like peptide 2 (GLP-2) exerts similar effects on ISCs during intestinal stress.^55^ The GLP-2 analog, teduglutide, promotes ISC expansion after intestinal resection,^56^ protects ISCs from radiation damage,^57^ and facilitates ISC repair during graft-versus-host disease.^58^ Notably, NT and GLP-2 exert similar trophic actions in the small intestine under basal and stress conditions. Both NT and GLP-2 induce mucosal growth and proliferation,^25^^,^^59^ inhibit apoptosis,^27^^,^^60^ and promote intestinal repair after inflammatory damage.^61^^,^^62^ GLP-2 also facilitates nutrient-regulated growth of the small intestine^63^ and prevents mucosal atrophy caused by total parenteral nutrition through regulation of AKT/GSK3β and WNT/β-catenin signaling.^64^ Our findings implicating NT in the regulation of ISC function through AKT/GSK3β and WNT/β-catenin signaling suggest that GLP-2 and NT exert analogous nutrient-dependent trophic functions through similar molecular mechanisms. Notably, a combination of NT and GLP-2 produced greater effects on intestinal growth in mice than either NT or GLP-2 alone.^65^

Previous work describing the trophic functions of NT in the intestine used exogenous NT.^32^ Thus, the current study examined the effects of endogenous NT on intestinal proliferation. Interestingly, exogenous NT elicits a more pronounced proliferative response in the proximal small-bowel mucosa relative to the distal small intestine.^25^^,^^26^ Our current findings indicate that endogenous NT has a more pronounced effect in the distal crypts, which is likely due to the localization of NT predominantly in the distal small intestine. These findings suggest that endogenous NT release from distal EE cells regulates the local ISC niche in a paracrine manner. Similar to NT, many other gut hormones are secreted from EE cells and are localized to discrete regions along the length of the stomach, small bowel, and colon.^66^ Given the important local effects of NT in the distal small intestine, it is interesting to speculate that other gut hormones may exert similar paracrine functions that influence the local microenvironment in which they are released.

Mihaylova et al^4^ showed that up-regulation of FAO, via PPARδ signaling and *Cpt1a* expression, is critical for enhanced ISC function after a 24-hour fast. In our study, loss of NT impaired fasting-induced ISC function without altering expression of *Cpt1a*, suggesting that the effect of NT on ISC function during nutrient deprivation is independent of FAO. Instead, our findings indicate that NT promotes ISC function by up-regulating WNT/β-catenin signaling and *Lgr5* mRNA expression in ISCs and progenitor cells. Because PPARδ is a WNT target,^67^ it is plausible that the increase in PPARδ signaling during fasting observed by us and others^4^ is a product of WNT/β-catenin activation. This is consistent with the observation that expression of *Lgr5* mRNA and WNT target genes is up-regulated in the crypts of human ileum after prolonged absence of enteral nutrients, despite a reduction in total ISC numbers.^68^ Together, these findings suggest that *Lgr5* expression is enhanced in ISCs during nutrient depletion, likely as a priming mechanism to allow for rapid proliferation upon the re-introduction of nutrients.^68^

Our findings establish a role for NT in positive regulation of WNT/β-catenin signaling in the small intestine. NT-mediated activation of the WNT/β-catenin pathway was associated with increased phosphorylation of AKT and GSK3β, whereas absence of NT decreased AKT and GSK3β phosphorylation. These findings corroborate the effects of NT in CRC cells, in which NT promotes phosphorylation of AKT and GSK3β and enhances cell proliferation.^31^ Consistent with studies showing WNT activation downstream of GSK3β phosphorylation,^40^, ^41^, ^42^ we found that NT up-regulates expression of active β-catenin and WNT target genes during fasting, suggesting that NT acts upstream of AKT and GSK3β to promote WNT/β-catenin signaling. Although we did not detect NT-dependent differences in AKT activity in fasted crypts, our findings are consistent with Richmond et al,^36^ showing nearly undetectable levels of p-AKT in intestinal crypts after a 48-hour fast. We speculate that the observed level of AKT activity during fasting is the minimum required for crypt maintenance and therefore is not inhibited further by loss of NT. Interestingly, loss of NT markedly reduced p-AKT and p-GSK3β expression in the crypts of ad libitum–fed mice without altering WNT pathway activation. We postulate that under nutrient-rich conditions, in which crypts are rapidly proliferating, the effect of NT on these growth-stimulating pathways serves mainly to contribute to crypt cell proliferation. Considering our observation that fasting reduces total GSK3β expression, the pool of GSK3β phosphorylated downstream of NT may become more critical in the regulation of WNT/β-catenin signaling during nutrient deprivation.

Although NT activates ERK1/2 signaling in numerous cancer cell lines in vitro,^69^ a role for NT on ERK1/2 activation in the small intestine has not been described previously. This study conclusively established NT as a positive regulator of ERK1/2 signaling in the small intestine in vivo. Exogenous NT activated, whereas NT deficiency attenuated, ERK1/2 phosphorylation in the small intestine. ERK1/2 controls cell-cycle progression and regulates cyclin D1 transcription, and proliferation of intestinal epithelial cells requires ERK1/2 activation.^70^, ^71^, ^72^, ^73^, ^74^ In agreement with these studies, NT deficiency significantly reduced cyclin D1 expression and progenitor cell proliferation in the distal crypts. Consistent with the observed pattern of AKT activity, the impact of NT deficiency on p-ERK1/2 and cyclin D1 was more pronounced under nutrient-rich conditions, further indicating that NT promotes proliferative signaling pathways, primarily under highly proliferative, nutrient-rich conditions. In agreement with this, reduced proliferation during nutrient-depletion coincided with decreased *Nt* mRNA expression, suggesting that impaired crypt cell proliferation may be due, in part, to decreased NT release. Attenuation of crypt cell proliferation noted in *Nt*^*-/-*^ mice was associated with increased crypt cell differentiation, as shown by the increase in cells stained by Alcian blue.^75^ Cyclin–cyclin-dependent kinase complexes regulate the switch from active cell proliferation to terminal differentiation, and cyclin D1 expression is correlated inversely with goblet cell differentiation in multiple mouse models.^76^, ^77^, ^78^ Thus, attenuation of cyclin D1 expression associated with the absence of NT may shift the balance from progenitor cell proliferation to terminal differentiation. Together, these data suggest that the trophic effects of NT in the small intestine are mediated, in part, by positive regulation of ERK1/2 signaling and cell-cycle progression.

In conclusion, our study defines a novel and dynamic function for NT in the maintenance of intestinal homeostasis that is highly dependent on nutrient status (Figure 7*H*). During periods of nutrient abundance (ie, fed state), NT activates the ERK1/2 signaling pathway to contribute to intestinal proliferation. However, during periods of nutrient deprivation (ie, starvation), NT signals through the WNT/β-catenin pathway to preserve ISC function. Given the critical importance of digestion and nutrient absorption and the central role intestinal hormones play in these life-sustaining processes, from a teleologic perspective, we postulate that gut hormones (eg, NT) serve multiple roles in the intestine and that these roles are dependent on nutrient abundance. In the fed state, hormones act predominantly in an endocrine fashion on distant organs to facilitate and ensure the digestion and absorption of ingested nutrients. However, with the scarcity of nutrients, these hormones can act locally to preserve and maintain ISC function. Importantly, our findings expand our current knowledge regarding the effects of intestinal hormones and suggest that, in addition to their known endocrine effects, certain intestinal hormones (eg, NT) function locally to preserve intestinal equilibrium during periods of nutrient deprivation.

## Materials and Methods

### Mice

All procedures were approved by the Institutional Animal Care and Use Committee of the University of Kentucky. *Nt*^*+/+*^, *Nt*^*-/-*^, Lgr5-EGFP-IRES-CreERT2^14^ (strain name: B6.129P2-Lgr5tm1(cre/ERT2)Cle/J, stock number: 008875; Jackson Laboratory, Bar Harbor, ME), and Lgr5-EGFP-IRES-CreERT2 *Nt*^*-/-*^ mice were maintained on a 14-hour light/10-hour dark cycle and provided with food and water ad libitum unless otherwise indicated. For fasting studies, mice were fasted for 48 hours beginning at 9 AM with ad libitum access to water only. Body weight was measured daily during the fasting period. Control mice were sex- and age-matched littermates fed standard chow ad libitum. Both male and female mice (age, 10–16 wk) were used for fasting studies.

### Crypt Isolation and Organoid Culture

Crypts were isolated from the proximal two thirds of the small intestine as previously described.^15^ Briefly, the small intestine was removed, opened longitudinally, washed with PBS, and cut into 3- to 5-mm fragments. Fragments were incubated in cold PBS containing 10 mmol/L EDTA for 30–60 minutes on ice at 4°C, followed by vortexing and filtration through a 70-μmol/L cell strainer. Isolated crypts were collected for RNA and protein extraction or organoid culture. For organoid culture, crypts were embedded in growth-factor reduced Matrigel (Corning, Corning, NY) and cultured in basal organoid media supplemented with 40 ng/mL epidermal growth factor (Peprotech, East Windsor, NJ), 200 ng/mL noggin (Peprotech), and 500 ng/mL R-spondin (Sino Biological, Beijing, China). Basal organoid media contained advanced Dulbecco’s modified Eagle medium/F12 (Gibco, Amarillo, TX) supplemented with 1 mol/L N-acetyl-L-cysteine (Sigma-Aldrich, St. Louis, MO), 1× N2 (Life Technologies, Carlsbad, CA), 1× B27 (Life Technologies), 1× penicillin/streptomycin (Sigma-Aldrich), 1× HEPES (Sigma-Aldrich), and 1× Glutamax (Sigma-Aldrich). For colony-formation assays, freshly isolated crypts were counted and plated in triplicate in 48-well plates. Organoid formation was quantified 3 days after initiation of cultures and normalized to the number of crypts plated per well at day 0.

### Organoid EdU Labeling

EdU labeling of organoids was achieved using the Click-iT EdU Cell Proliferation Kit for Imaging (Invitrogen, Carlsbad, CA) according to the manufacturer’s instructions. EdU-positive cells were quantified using Nikon (Melville, NY) software and expressed as the Pearson coefficient for colocalization between 4',6-diamidino-2-phenylindole and EdU. Colocalization was measured in 20–30 organoids from 3 to 4 different mice per group.

### Real-time qPCR and In Situ Hybridization

Total RNA was isolated from crypts or organoids using the RNeasy Mini Kit (Qiagen, Germantown, MD) according to the manufacturer’s instructions. cDNA was synthesized from equal concentrations of total RNA using the High-Capacity cDNA Reverse Transcription Kit (Applied Biosystems, Waltham, MA). Real-time qPCR was performed using a TaqMan Gene Expression Master Mix (ThermoFisher, Waltham, MA) and TaqMan probes for *Tcf7*, *c-Myc*, *Lgr5*, *Ascl2*, *Olfm4*, *Cpt1a*, *Hmgcs2*, *Nt*, and *Actb* (ThermoFisher) according to the manufacturer's protocol. Relative mRNA expression was calculated using the comparative delta-delta Ct method.

Single-molecule in situ hybridization was performed using the Advanced Cell Diagnostics RNAscope 2.0 HD Detection Kit (ACD Bio, Newark, CA) with mouse *Lgr5 Mm-Lgr5* (ref: 312171). Antigen retrieval was performed for 15 minutes and peroxidase pretreatment was performed for 30 minutes. Stained sections were imaged using an Aperio ScanScope XT (Vista, CA) slide scanner at 20× and *Lgr5* expression was analyzed using HALO software (Indica Labs, Albuquerque, NM).

### RNA Sequencing, Differential Expression, and GSEA

Isolated RNA quality and quantity was assessed using the Agilent Bioanalyzer 2100 RNA Nano chip (La Jolla, CA. RNA sequencing libraries were prepared using KAPA RNA Hyper + RiboErase HMR (Roche, Indianapolis, IN). The manufacturer’s protocols were used to sequence ribosomal RNA-depleted libraries at 1 × 100 single-end read on an Illumina HiSeq 2500 (Hayward, CA) in rapid mode, to an average depth of 32 million single-end reads per sample. The data were stored as FASTQ files. Sequencing reads were trimmed and filtered using Trimmomatic (V0.39) to remove adapters and low-quality reads.^79^ Reads were mapped to Ensembl GRCm38 (release 100) transcripts annotation using RSEM.^80^ Differential expression analyses were performed using the R package edgeR.^81^ Significantly up-regulated/down-regulated genes were determined as false-discovery rate–adjusted *P* value < .05. GSEA was performed using GSEA software.^82^

### Flow Cytometry

Fresh intestinal crypts or organoids were incubated in TrypLE Express (Invitrogen) for 15 minutes at 37°C and then passed through a 23G needle to achieve a single-cell suspension.^15^ Cells were washed in PBS, resuspended in PBS containing 1 mmol/L EDTA, 25 mmol/L HEPES, 1% fetal bovine serum, and DNase (1 μg/mL), and filtered through a 40-μm cell strainer. Cells were labeled with CD11b-PE, CD31-PE, CD45-PE, CD133-PE, and epithelial cell adhesion molecule (EPCAM)-APC (BioLegend, San Diego, CA), and sorted with a BD FACS Aria II SORP (Franklin Lakes, NJ) cell sorter.^15^ Lgr5-EGFP^+^/EPCAM^+^/CD11b^-^ CD31^-^ CD45^-^ CD133^-^ cells were quantified using FlowJo (v. 10, Franklin Lakes, NJ) software.^15^ Dead cells were excluded from analysis using propidium iodide (50 μg/mL) (Abcam, Boston, MA).

### Immunohistochemistry and Immunoblotting

For histologic analysis, the small intestine (including duodenum, jejunum, and ileum) was divided into thirds and the proximal and distal thirds were fixed overnight in 10% neutral buffered formalin, paraffin-embedded, and sectioned. Antigen retrieval was performed with Tris-EDTA buffer, pH 7.8, at 95°C for 64 minutes (standard cell conditioning solution 1). Staining was performed on the Ventana Autostainer with Ki67 (16667, 1:200 for 1 h at 37°C; Abcam). Alcian blue (a-5268; Sigma-Aldrich) staining was performed manually with 1% Alcian blue solution in 3% glacial acetic acid. Ventana OmniMap anti-rabbit horseradish peroxidase and 3,3′-diaminobenzidine tetra hydrochloride were used for visualization per the manufacturer’s instructions. Stained sections were imaged using an Aperio ScanScope XT slide scanner at 20× and analyzed using HALO software (Indica Labs). Ki67^+^ and Alcian blue^+^ cells were counted in 50 crypts per section in the proximal and distal small intestine from at least 5 mice per group.

For Western blot, crypts or organoids were lysed with lysis buffer (Cell Signaling Technology, Danvers, MA) containing 1 mmol/L phenylmethylsulfonyl fluoride. Equal amounts of protein were resolved on 4%–12% NuPAGE BisTris gels (Invitrogen) and electrophoretically transferred to polyvinylidene difluoride membranes. The following antibodies were used for Western blot: p-ERK (#9101; CST), ERK (#9102; CST), GSK3β (#12456; CST), p-GSK3β (#9322; CST), LRP6 (#3395; CST), p-LRP6 (#2568; CST), AKT (#4691; CST), p-AKT (#4058; CST), active β-catenin (#8814; CST), total β-catenin (#8480; CST), and β-actin (A5316; Sigma-Aldrich). Membranes were developed with the iBright FL1500 Imaging System (Invitrogen). Band intensity was measured using iBright Analysis Software (ThermoFisher), using β-actin or total protein as loading controls.

### Drosophila Studies

Constitutive expression of NT in gut EE cells was achieved by cloning the gut EE cell-specific TK promoter (2.0 kb) into attB-UAST lacking Gal4 binding sites followed by insertion of full-length NT cDNA.^48^^,^^83^ The resulting plasmid (TK-NT) was inserted at the VK5 attP locus. *esg*-Gal4-UAS-GFP/Cyo, +/TM6B and *esg*-Gal4-UAS-GFP/Cyo, and TK-NT/TM6B flies were fed standard fly food (cornmeal-yeast) or a LED.^50^^,^^51^ Briefly, flies (5 days after emerging) were maintained on standard fly food for an additional 5 days, split into 2 groups, and fed either standard fly food or a LED fly food (diet composition in 100 mL media: 4 g yeast extract, 4 g sucrose, 5.2 g cornmeal, and 1.5 g agar) for an additional 5 days. Midguts were dissected, fixed in 4% formaldehyde in PBS for 20 minutes, permeabilized in 0.1% Triton X-100 (ThermoFisher, Waltham, MA) in PBS, and processed for either immunohistochemistry using an α-GFP (Clontech, Fitchburg, WI) primary antibody or lipid staining using Nile Red. Fluorescence signals were acquired on an Olympus (CEnter Valley, PA) confocal microscope and images were processed with an Olympus FV10-ASW version 3.1b. ISCs (100–700) were quantified in the midgut of 8–15 flies per group and normalized to the area of midgut captured per image.

### Statistical Analysis

All experiments were performed at least 3 independent times and all sample numbers (n) indicate biological replicates. For colony formation assays, 3–4 wells of organoids from 5–6 mice per group were analyzed. All quantitative data on real-time qPCR for genes, Western blot and immunohistochemistry for proteins, organoid labeling, and fluorescence levels for ISC quantification were summarized using bar graphs with means and SDs. Comparisons across experimental factors, which includes a combination of genotype and diet, were analyzed using 1-way analysis of variance or repeated-measures linear mixed model to account for repeated measures within mice. Adjustment for multiple pairwise comparisons was performed within the model using the Holm’s *P* value stepdown method. Assessment of model assumptions was performed, and data were log-transformed as necessary for optimal fit of the model. Statistical analyses were performed using the SAS system 9.4 (Cary, NC). Bioinformatics methods for differential analysis were performed using the R package as described earlier.

All authors had access to the study data and reviewed and approved the final manuscript.

## References

[1] van der Flier L.G., Clevers H. (2009). Stem cells, self-renewal, and differentiation in the intestinal epithelium. Annu Rev Physiol.

[2] Baulies A., Angelis N., Li V.S.W. (2020). Hallmarks of intestinal stem cells. Development.

[3] Shaw D., Gohil K., Basson M.D. (2012). Intestinal mucosal atrophy and adaptation. World J Gastroenterol.

[4] Mihaylova M.M., Cheng C.W., Cao A.Q., Tripathi S., Mana M.D., Bauer-Rowe K.E., Abu-Remaileh M., Clavain L., Erdemir A., Lewis C.A., Freinkman E., Dickey A.S., La Spada A.R., Huang Y., Bell G.W., Deshpande V., Carmeliet P., Katajisto P., Sabatini D.M., Yilmaz O.H. (2018). Fasting activates fatty acid oxidation to enhance intestinal stem cell function during homeostasis and aging. Cell Stem Cell.

[5] Igarashi M., Guarente L. (2016). mTORC1 and SIRT1 cooperate to foster expansion of gut adult stem cells during calorie restriction. Cell.

[6] Papa S., Choy P.M., Bubici C. (2019). The ERK and JNK pathways in the regulation of metabolic reprogramming. Oncogene.

[7] Sethi J.K., Vidal-Puig A. (2010). Wnt signalling and the control of cellular metabolism. Biochem J.

[8] Perochon J., Carroll L.R., Cordero J.B. (2018). Wnt signalling in intestinal stem cells: lessons from mice and flies. Genes (Basel).

[9] Pinto D., Gregorieff A., Begthel H., Clevers H. (2003). Canonical Wnt signals are essential for homeostasis of the intestinal epithelium. Genes Dev.

[10] Logan C.Y., Nusse R. (2004). The Wnt signaling pathway in development and disease. Annu Rev Cell Dev Biol.

[11] Reya T., Clevers H. (2005). Wnt signalling in stem cells and cancer. Nature.

[12] Sato T., van Es J.H., Snippert H.J., Stange D.E., Vries R.G., van den Born M., Barker N., Shroyer N.F., van de Wetering M., Clevers H. (2011). Paneth cells constitute the niche for Lgr5 stem cells in intestinal crypts. Nature.

[13] Valenta T., Degirmenci B., Moor A.E., Herr P., Zimmerli D., Moor M.B., Hausmann G., Cantu C., Aguet M., Basler K. (2016). Wnt ligands secreted by subepithelial mesenchymal cells are essential for the survival of intestinal stem cells and gut homeostasis. Cell Rep.

[14] Barker N., van Es J.H., Kuipers J., Kujala P., van den Born M., Cozijnsen M., Haegebarth A., Korving J., Begthel H., Peters P.J., Clevers H. (2007). Identification of stem cells in small intestine and colon by marker gene Lgr5. Nature.

[15] Beyaz S., Mana M.D., Roper J., Kedrin D., Saadatpour A., Hong S.J., Bauer-Rowe K.E., Xifaras M.E., Akkad A., Arias E., Pinello L., Katz Y., Shinagare S., Abu-Remaileh M., Mihaylova M.M., Lamming D.W., Dogum R., Guo G., Bell G.W., Selig M., Nielsen G.P., Gupta N., Ferrone C.R., Deshpande V., Yuan G.C., Orkin S.H., Sabatini D.M., Yilmaz O.H. (2016). High-fat diet enhances stemness and tumorigenicity of intestinal progenitors. Nature.

[16] Anagnostou S.H., Shepherd P.R. (2008). Glucose induces an autocrine activation of the Wnt/beta-catenin pathway in macrophage cell lines. Biochem J.

[17] Lu V.B., Gribble F.M., Reimann F. (2021). Nutrient-induced cellular mechanisms of gut hormone secretion. Nutrients.

[18] Evers B.M., Ehrenfried J.A., Wang X., Townsend C.M., Thompson J.C. (1994). Temporal-specific and spatial-specific patterns of neurotensin gene expression in the small bowel. Am J Physiol.

[19] Barber D.L., Cacace A.M., Raucci D.T., Ganz M.B. (1991). Fatty acids stereospecifically stimulate neurotensin release and increase [Ca2+]i in enteric endocrine cells. Am J Physiol.

[20] Li J., Song J., Zaytseva Y.Y., Liu Y., Rychahou P., Jiang K., Starr M.E., Kim J.T., Harris J.W., Yiannikouris F.B., Katz W.S., Nilsson P.M., Orho-Melander M., Chen J., Zhu H., Fahrenholz T., Higashi R.M., Gao T., Morris A.J., Cassis L.A., Fan T.W., Weiss H.L., Dobner P.R., Melander O., Jia J., Evers B.M. (2016). An obligatory role for neurotensin in high-fat-diet-induced obesity. Nature.

[21] Kim J.T., Weiss H.L., Evers B.M. (2017). Diverse expression patterns and tumorigenic role of neurotensin signaling components in colorectal cancer cells. Int J Oncol.

[22] Boules M., Li Z., Smith K., Fredrickson P., Richelson E. (2013). Diverse roles of neurotensin agonists in the central nervous system. Front Endocrinol (Lausanne).

[23] Zhao D., Pothoulakis C. (2006). Effects of NT on gastrointestinal motility and secretion, and role in intestinal inflammation. Peptides.

[24] Christou N., Blondy S., David V., Verdier M., Lalloue F., Jauberteau M.O., Mathonnet M., Perraud A. (2020). Neurotensin pathway in digestive cancers and clinical applications: an overview. Cell Death Dis.

[25] Wood J.G., Hoang H.D., Bussjaeger L.J., Solomon T.E. (1988). Neurotensin stimulates growth of small intestine in rats. Am J Physiol.

[26] Izukura M., Evers B.M., Parekh D., Yoshinaga K., Uchida T., Townsend C.M., Thompson J.C. (1992). Neurotensin augments intestinal regeneration after small bowel resection in rats. Ann Surg.

[27] Devader C., Beraud-Dufour S., Coppola T., Mazella J. (2013). The anti-apoptotic role of neurotensin. Cells.

[28] Kim J.T., Liu C., Zaytseva Y.Y., Weiss H.L., Townsend C.M., Evers B.M. (2015). Neurotensin, a novel target of Wnt/beta-catenin pathway, promotes growth of neuroendocrine tumor cells. Int J Cancer.

[29] Xiao H., Zeng Y., Wang Q., Wei S., Zhu X. (2017). A novel positive feedback loop between NTSR1 and Wnt/beta-catenin contributes to tumor growth of glioblastoma. Cell Physiol Biochem.

[30] Ye Y., Long X., Zhang L., Chen J., Liu P., Li H., Wei F., Yu W., Ren X., Yu J. (2016). NTS/NTR1 co-expression enhances epithelial-to-mesenchymal transition and promotes tumor metastasis by activating the Wnt/beta-catenin signaling pathway in hepatocellular carcinoma. Oncotarget.

[31] Wang Q., Zhou Y., Evers B.M. (2006). Neurotensin phosphorylates GSK-3alpha/beta through the activation of PKC in human colon cancer cells. Neoplasia.

[32] Evers B.M. (2006). Neurotensin and growth of normal and neoplastic tissues. Peptides.

[33] Meloche S., Pouyssegur J. (2007). The ERK1/2 mitogen-activated protein kinase pathway as a master regulator of the G1- to S-phase transition. Oncogene.

[34] Beumer J., Clevers H. (2016). Regulation and plasticity of intestinal stem cells during homeostasis and regeneration. Development.

[35] Gerdes J., Schwab U., Lemke H., Stein H. (1983). Production of a mouse monoclonal antibody reactive with a human nuclear antigen associated with cell proliferation. Int J Cancer.

[36] Richmond C.A., Shah M.S., Deary L.T., Trotier D.C., Thomas H., Ambruzs D.M., Jiang L., Whiles B.B., Rickner H.D., Montgomery R.K., Tovaglieri A., Carlone D.L., Breault D.T. (2015). Dormant intestinal stem cells are regulated by PTEN and nutritional status. Cell Rep.

[37] Evers B.M., Rajaraman S., Chung D.H., Townsend C.M., Wang X., Graves K., Thompson J.C. (1993). Differential expression of the neurotensin gene in the developing rat and human gastrointestinal tract. Am J Physiol.

[38] Krausova M., Korinek V. (2014). Wnt signaling in adult intestinal stem cells and cancer. Cell Signal.

[39] Gao C., Xiao G., Hu J. (2014). Regulation of Wnt/beta-catenin signaling by posttranslational modifications. Cell Biosci.

[40] Tejeda-Munoz N., Robles-Flores M. (2015). Glycogen synthase kinase 3 in Wnt signaling pathway and cancer. IUBMB Life.

[41] Cross D.A., Alessi D.R., Cohen P., Andjelkovich M., Hemmings B.A. (1995). Inhibition of glycogen synthase kinase-3 by insulin mediated by protein kinase B. Nature.

[42] Fukumoto S., Hsieh C.M., Maemura K., Layne M.D., Yet S.F., Lee K.H., Matsui T., Rosenzweig A., Taylor W.G., Rubin J.S., Perrella M.A., Lee M.E. (2001). Akt participation in the Wnt signaling pathway through Dishevelled. J Biol Chem.

[43] Gully D., Canton M., Boigegrain R., Jeanjean F., Molimard J.C., Poncelet M., Gueudet C., Heaulme M., Leyris R., Brouard A., Pelaprat D., Labbe-Jullie C., Mazella J., Soubrie P., Maffrand J.-P., Rostene W., Kitabgi P., Le Fur G. (1993). Biochemical and pharmacological profile of a potent and selective nonpeptide antagonist of the neurotensin receptor. Proc Natl Acad Sci U S A.

[44] Spit M., Koo B.K., Maurice M.M. (2018). Tales from the crypt: intestinal niche signals in tissue renewal, plasticity and cancer. Open Biol.

[45] Salic A., Mitchison T.J. (2008). A chemical method for fast and sensitive detection of DNA synthesis in vivo. Proc Natl Acad Sci U S A.

[46] Zwick R.K., Ohlstein B., Klein O.D. (2019). Intestinal renewal across the animal kingdom: comparing stem cell activity in mouse and Drosophila. Am J Physiol Gastrointest Liver Physiol.

[47] Loza-Coll M.A., Southall T.D., Sandall S.L., Brand A.H., Jones D.L. (2014). Regulation of Drosophila intestinal stem cell maintenance and differentiation by the transcription factor Escargot. EMBO J.

[48] Amcheslavsky A., Song W., Li Q., Nie Y., Bragatto I., Ferrandon D., Perrimon N., Ip Y.T. (2014). Enteroendocrine cells support intestinal stem-cell-mediated homeostasis in Drosophila. Cell Rep.

[49] Song W., Cheng D., Hong S., Sappe B., Hu Y., Wei N., Zhu C., O'Connor M.B., Pissios P., Perrimon N. (2017). Midgut-derived activin regulates glucagon-like action in the fat body and glycemic control. Cell Metab.

[50] Min K.J., Flatt T., Kulaots I., Tatar M. (2007). Counting calories in Drosophila diet restriction. Exp Gerontol.

[51] Skorupa D.A., Dervisefendic A., Zwiener J., Pletcher S.D. (2008). Dietary composition specifies consumption, obesity, and lifespan in Drosophila melanogaster. Aging Cell.

[52] Mana M.D., Kuo E.Y., Yilmaz O.H. (2017). Dietary regulation of adult stem cells. Curr Stem Cell Rep.

[53] Tang K.H., Ma S., Lee T.K., Chan Y.P., Kwan P.S., Tong C.M., Ng I.O., Man K., To K.F., Lai P.B., Lo C.M., Guan X.Y., Chan K.W. (2012). CD133(+) liver tumor-initiating cells promote tumor angiogenesis, growth, and self-renewal through neurotensin/interleukin-8/CXCL1 signaling. Hepatology.

[54] Zhou J., Yi L., Ouyang Q., Xu L., Cui H., Xu M. (2014). Neurotensin signaling regulates stem-like traits of glioblastoma stem cells through activation of IL-8/CXCR1/STAT3 pathway. Cell Signal.

[55] Rowland K.J., Brubaker P.L. (2011). The "cryptic" mechanism of action of glucagon-like peptide-2. Am J Physiol Gastrointest Liver Physiol.

[56] Garrison A.P., Dekaney C.M., von Allmen D.C., Lund P.K., Henning S.J., Helmrath M.A. (2009). Early but not late administration of glucagon-like peptide-2 following ileo-cecal resection augments putative intestinal stem cell expansion. Am J Physiol Gastrointest Liver Physiol.

[57] Booth C., Booth D., Williamson S., Demchyshyn L.L., Potten C.S. (2004). Teduglutide ([Gly2]GLP-2) protects small intestinal stem cells from radiation damage. Cell Prolif.

[58] Norona J., Apostolova P., Schmidt D., Ihlemann R., Reischmann N., Taylor G., Kohler N., de Heer J., Heeg S., Andrieux G., Siranosian B.A., Schmitt-Graeff A., Pfeifer D., Catalano A., Frew I.J., Proietti M., Grimbacher B., Bulashevska A., Bhatt A.S., Brummer T., Clauditz T., Zabelina T., Kroeger N., Blazar B.R., Boerries M., Ayuk F., Zeiser R. (2020). Glucagon-like peptide 2 for intestinal stem cell and Paneth cell repair during graft-versus-host disease in mice and humans. Blood.

[59] Drucker D.J., Erlich P., Asa S.L., Brubaker P.L. (1996). Induction of intestinal epithelial proliferation by glucagon-like peptide 2. Proc Natl Acad Sci U S A.

[60] Tsai C.H., Hill M., Asa S.L., Brubaker P.L., Drucker D.J. (1997). Intestinal growth-promoting properties of glucagon-like peptide-2 in mice. Am J Physiol.

[61] Drucker D.J., Yusta B., Boushey R.P., DeForest L., Brubaker P.L. (1999). Human [Gly2]GLP-2 reduces the severity of colonic injury in a murine model of experimental colitis. Am J Physiol.

[62] Brun P., Mastrotto C., Beggiao E., Stefani A., Barzon L., Sturniolo G.C., Palu G., Castagliuolo I. (2005). Neuropeptide neurotensin stimulates intestinal wound healing following chronic intestinal inflammation. Am J Physiol Gastrointest Liver Physiol.

[63] Shin E.D., Estall J.L., Izzo A., Drucker D.J., Brubaker P.L. (2005). Mucosal adaptation to enteral nutrients is dependent on the physiologic actions of glucagon-like peptide-2 in mice. Gastroenterology.

[64] Feng Y., Demehri F.R., Xiao W., Tsai Y.H., Jones J.C., Brindley C.D., Threadgill D.W., Holst J.J., Hartmann B., Barrett T.A., Teitelbaum D.H., Dempsey P.J. (2017). Interdependency of EGF and GLP-2 signaling in attenuating mucosal atrophy in a mouse model of parenteral nutrition. Cell Mol Gastroenterol Hepatol.

[65] Litvak D.A., Evers B.M., Hellmich M.R., Townsend C.M. (1999). Enterotrophic effects of glucagon-like peptide 2 are enhanced by neurotensin. J Gastrointest Surg.

[66] Svendsen B., Pedersen J., Albrechtsen N.J., Hartmann B., Torang S., Rehfeld J.F., Poulsen S.S., Holst J.J. (2015). An analysis of cosecretion and coexpression of gut hormones from male rat proximal and distal small intestine. Endocrinology.

[67] Sidrat T., Rehman Z.U., Joo M.D., Lee K.L., Kong I.K. (2021). Wnt/beta-catenin pathway-mediated PPARdelta expression during embryonic development differentiation and disease. Int J Mol Sci.

[68] Wieck M.M., Schlieve C.R., Thornton M.E., Fowler K.L., Isani M., Grant C.N., Hilton A.E., Hou X., Grubbs B.H., Frey M.R., Grikscheit T.C. (2017). Prolonged absence of mechanoluminal stimulation in human intestine alters the transcriptome and intestinal stem cell niche. Cell Mol Gastroenterol Hepatol.

[69] Nikolaou S., Qiu S., Fiorentino F., Simillis C., Rasheed S., Tekkis P., Kontovounisios C. (2020). The role of neurotensin and its receptors in non-gastrointestinal cancers: a review. Cell Commun Signal.

[70] Chambard J.C., Lefloch R., Pouyssegur J., Lenormand P. (2007). ERK implication in cell cycle regulation. Biochim Biophys Acta.

[71] Lavoie J.N., L'Allemain G., Brunet A., Muller R., Pouyssegur J. (1996). Cyclin D1 expression is regulated positively by the p42/p44MAPK and negatively by the p38/HOGMAPK pathway. J Biol Chem.

[72] Boucher M.J., Jean D., Vezina A., Rivard N. (2004). Dual role of MEK/ERK signaling in senescence and transformation of intestinal epithelial cells. Am J Physiol Gastrointest Liver Physiol.

[73] Daksis J.I., Lu R.Y., Facchini L.M., Marhin W.W., Penn L.J. (1994). Myc induces cyclin D1 expression in the absence of de novo protein synthesis and links mitogen-stimulated signal transduction to the cell cycle. Oncogene.

[74] Zeiser R. (2014). Trametinib. Recent results. Cancer Res.

[75] Greco V., Lauro G., Fabbrini A., Torsoli A. (1967). Histochemistry of the colonic epithelial mucins in normal subjects and in patients with ulcerative colitis. A qualitative and histophotometric investigation. Gut.

[76] Ruijtenberg S., van den Heuvel S. (2016). Coordinating cell proliferation and differentiation: antagonism between cell cycle regulators and cell type-specific gene expression. Cell Cycle.

[77] Hulit J., Wang C., Li Z., Albanese C., Rao M., Di Vizio D., Shah S., Byers S.W., Mahmood R., Augenlicht L.H., Russell R., Pestell R.G. (2004). Cyclin D1 genetic heterozygosity regulates colonic epithelial cell differentiation and tumor number in ApcMin mice. Mol Cell Biol.

[78] Li C., Zhou Y., Rychahou P., Weiss H.L., Lee E.Y., Perry C.L., Barrett T.A., Wang Q., Evers B.M. (2020). SIRT2 contributes to the regulation of intestinal cell proliferation and differentiation. Cell Mol Gastroenterol Hepatol.

[79] Bolger A.M., Lohse M., Usadel B. (2014). Trimmomatic: a flexible trimmer for Illumina sequence data. Bioinformatics.

[80] Li B., Dewey C.N. (2011). RSEM: accurate transcript quantification from RNA-seq data with or without a reference genome. BMC Bioinformatics.

[81] Robinson M.D., McCarthy D.J., Smyth G.K. (2010). edgeR: a Bioconductor package for differential expression analysis of digital gene expression data. Bioinformatics.

[82] Subramanian A., Tamayo P., Mootha V.K., Mukherjee S., Ebert B.L., Gillette M.A., Paulovich A., Pomeroy S.L., Golub T.R., Lander E.S., Mesirov J.P. (2005). Gene set enrichment analysis: a knowledge-based approach for interpreting genome-wide expression profiles. Proc Natl Acad Sci U S A.

[83] Song W., Veenstra J.A., Perrimon N. (2014). Control of lipid metabolism by tachykinin in Drosophila. Cell Rep.

